# BinaRena: a dedicated interactive platform for human-guided exploration and binning of metagenomes

**DOI:** 10.1186/s40168-023-01625-8

**Published:** 2023-08-19

**Authors:** Michael J. Pavia, Abhinav Chede, Zijun Wu, Hinsby Cadillo-Quiroz, Qiyun Zhu

**Affiliations:** 1 School of Life Sciences, Arizona State University, Tempe, AZ USA; 2 Biodesign Center for Fundamental and Applied Microbiomics, Arizona State University, Tempe, AZ USA; 3 Biodesign Swette Center for Environmental Biotechnology, Arizona State University, Tempe, AZ USA; 4 Center for Biophysics and Quantitative Biology, University of Illinois at Urbana-Champaign, Urbana, IL USA

**Keywords:** Metagenomics, Human factor, Contigs, Binning, Visualization, Interactive, JavaScript

## Abstract

**Background:**

Exploring metagenomic contigs and “binning” them into metagenome-assembled genomes (MAGs) are essential for the delineation of functional and evolutionary guilds within microbial communities. Despite the advances in automated binning algorithms, their capabilities in recovering MAGs with accuracy and biological relevance are so far limited. Researchers often find that human involvement is necessary to achieve representative binning results. This manual process however is expertise demanding and labor intensive, and it deserves to be supported by software infrastructure.

**Results:**

We present BinaRena, a comprehensive and versatile graphic interface dedicated to aiding human operators to explore metagenome assemblies via customizable visualization and to associate contigs with bins. Contigs are rendered as an interactive scatter plot based on various data types, including sequence metrics, coverage profiles, taxonomic assignments, and functional annotations. Various contig-level operations are permitted, such as selection, masking, highlighting, focusing, and searching. Binning plans can be conveniently edited, inspected, and compared visually or using metrics including silhouette coefficient and adjusted Rand index. Completeness and contamination of user-selected contigs can be calculated in real time.

In demonstration of BinaRena’s usability, we show that it facilitated biological pattern discovery, hypothesis generation, and bin refinement in a complex tropical peatland metagenome. It enabled isolation of pathogenic genomes within closely related populations from the gut microbiota of diarrheal human subjects. It significantly improved overall binning quality after curating results of automated binners using a simulated marine dataset.

**Conclusions:**

BinaRena is an installation-free, dependency-free, client-end web application that operates directly in any modern web browser, facilitating ease of deployment and accessibility for researchers of all skill levels. The program is hosted at https://github.com/qiyunlab/binarena, together with documentation, tutorials, example data, and a live demo. It effectively supports human researchers in intuitive interpretation and fine tuning of metagenomic data.

Video Abstract

**Supplementary Information:**

The online version contains supplementary material available at 10.1186/s40168-023-01625-8.

## Background

The rapid advancement in high-throughput sequencing technologies has led to the discovery of an enormous amount of new biodiversity from uncultivated microbial populations [1]. Extracting population genomes from heterogenous microbial communities is essential to understand the contribution of defined microbial lineages to host and environmental processes. Genome-resolved metagenomic studies have provided valuable insight into understanding microbial links to biogeochemistry [2–5], connections to human health and disease [6–8], and discovery of novel microbial groups [9, 10]. Exploration of such datasets can quickly become cumbersome [11–15], comprising hundreds of metagenome-assembled genomes (MAGs) with associated sequence characteristics, functional potential, and abundance across samples.

The building blocks of this comprehensive data are contigs, the minimum units of a genomic sequence derived from the assembly of metagenomic reads. Using characteristics such as nucleotide composition and sequencing depth, similar contigs can be grouped into “bins” representative of microbial populations’ genomes (i.e., MAGs). Despite the wealth of automatic binning tools [16–19], an intermediate step which can contextualize multiple layers of user-specified information for inspection of contig-to-bin assignment is necessary for reliable conclusions to be made. This human-guided step can greatly improve the quality of bins and subsequent inferences made from the contained biological information [1, 20–22]. This is because human brains are highly effective in pattern recognition [23], which was only recently challenged by algorithms in limited tasks [24], and this ability can be further enhanced by a priori knowledge of the biological systems. It has been accepted that exploratory data analysis [25], as characterized by heavy employment of data visualization and human involvement, is essential for understanding complex datasets, removing noise, discovering patterns, and generating hypotheses [26], and this cannot be replaced by any uniform algorithmic workflow.

Therefore, software infrastructure that helps human researchers in exploring metagenomic assemblies and defining bins (MAGs) is much needed [27]. Multiple tools have been developed to provide interactive visualization of metagenomes [28–32] (reviewed below), which can facilitate this process. However, few are explicitly designed with the goal of maximizing human productivity. Most tools constrain usability either through computational skill thresholds, or a relatively inflexible workflow, or a lack of study-specific customizable features.

To address this gap, we present BinaRena (“bin arena”), a comprehensive, highly customizable interactive graphical interface dedicated to human-guided exploration and binning of metagenomes. A visual representation of contigs is rendered as a scatter plot, displaying flexible types of data such as sequence metrics, coverage profiles, *k*-mer frequency, taxonomic assignment, feature annotation, existing binning outputs, and other metrics appropriate to the researcher. Integration of multiple layers of contig characteristics can aid delineation of microbial community members and improve overall binning results. The BinaRena program is free of installation, dependency, and a web server, making it exceptionally convenient for deployment and use. Licensed under BSD-3-clause, BinaRena’s source code is hosted at https://github.com/qiyunlab/binarena, together with comprehensive documentation, tutorials, example data, and a fully functional live demo.

To demonstrate BinaRena’s functionality and how it improves microbiome research, we analyzed one synthetic and two real-world metagenomic study cases. Specifically, we (1) analyzed the first metagenome available of a complex open tropical peatland from Maquia (MAQ) within the Pastaza-Marañón Foreland Basin, a globally important carbon reservoir in the Amazon, (2) reanalyzed metagenomes confounded by multiple pathogens from fecal samples of traveler’s diarrhea (TD) patients [33], and (3) quantified the systematic improvement of binning results using the gold standard CAMI2 marine dataset [19]. We show that BinaRena significantly facilitated pattern discovery, hypothesis generation, strain-level isolation, and bin refinement that were otherwise not achievable or overlooked by automatic workflows.

## Implementation

### Design and functionality of BinaRena

BinaRena is an installation-free, client-end web application. The user may simply double click “BinaRena.html” in the downloaded package to launch the program, which is literally a single webpage running in the user’s web browser, and does not require a web server running in the backend. In this sense, it is analogous to bioinformatics programs like Krona [34] and EMPeror [35]. BinaRena eliminates the need to execute a script for webpage construction, allowing users to simply drag and drop data files into the browser window to load them. This design minimizes the efforts for deployment and preparation, especially for nontechnical users, and computer systems with restrictions. The BinaRena program is written in pure JavaScript, without using any third-party frameworks or libraries. This ensures the program’s flexibility in behavior and functionality and allows the developing team to optimize the code for improved performance in rendering and calculation, which is important for handling modern metagenomic datasets, which usually contain tens to hundreds of thousands of contigs and many properties.

**Fig. 1:**
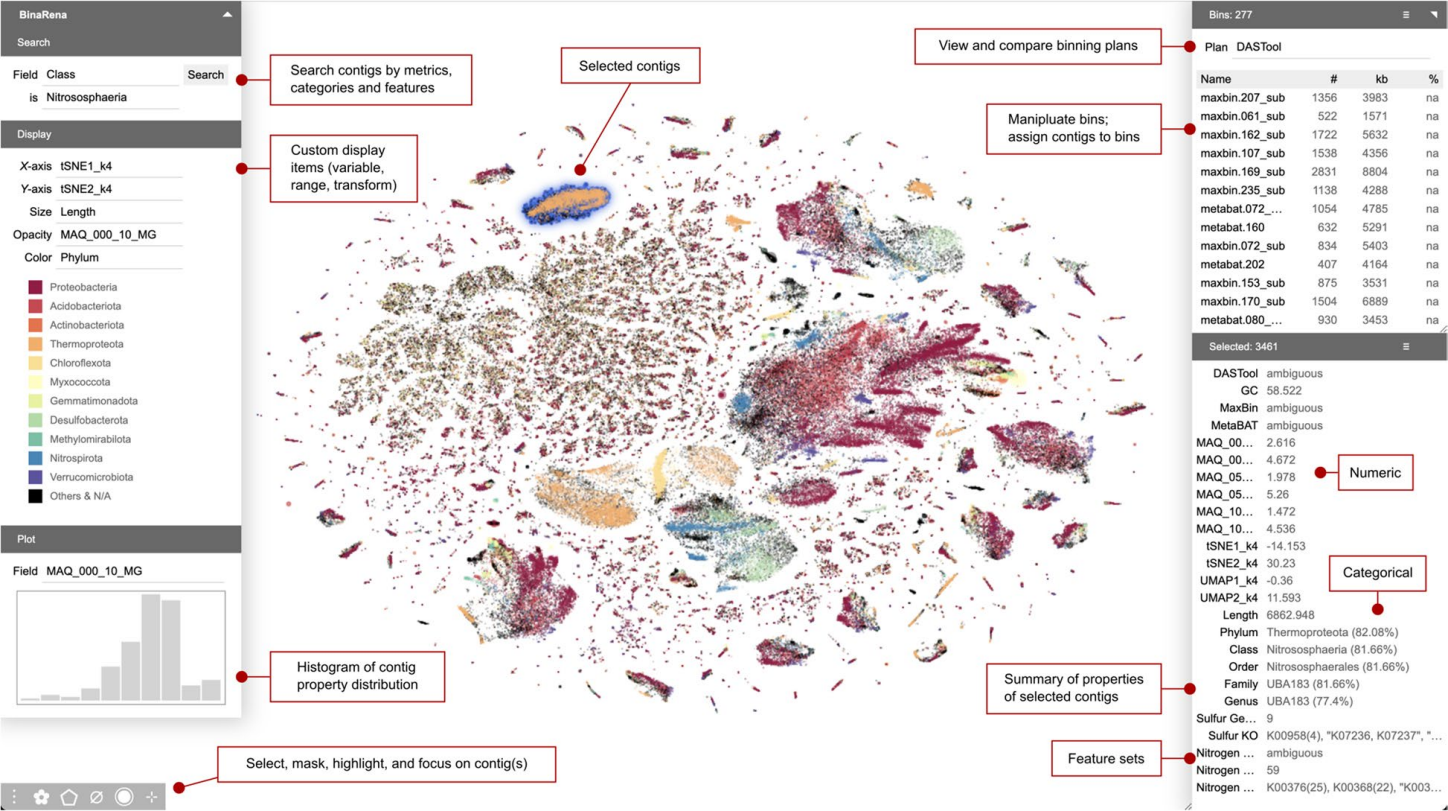
A screenshot of the main interface of BinaRena. The program is displaying the MAQ dataset, consisting of 262,705 contigs obtained from a co-assembly of six tropical peatland metagenomes. *X*- and *y*-axes represent t-SNE embeddings based on tetranucleotide frequencies. Marker size (radius) is proportional to the cube root of contig length. Marker opacity is proportional to the logarithm of sequencing depth (coverage) in one sample. Colors are assigned to the 10 most frequent phyla binned from DASTool. A binning plan consisting of 277 bins pre-computed by DASTool is loaded in the program, allowing the user to explore and manipulate individual bins by adding/removing individual contigs. A spatially distinct cluster of 3461 contigs putatively representing multiple Nitrososphaeria MAGs is currently selected by the user. The properties of the selected contigs are summarized in a side panel. The distribution of coverage is displayed as a histogram. Red-edged text boxes indicate functional components of the BinaRena interface

The main workspace of BinaRena is an interactive scatter plot, with data points representing contigs from an assembly (Fig. 1). The plot appearance is defined by five aesthetics: *x*- and *y*-axes, size, opacity, and color, each of which can be customized in the interface based on user-provided data that are relevant in delineating or relating contigs. For example, plotting GC% by coverage, comparing per-sample abundance profiles, and *k*-mer frequency-based dimensionality reduction are all helpful for contig clustering [28, 32, 36]. Reference-based properties such as taxonomic assignment and functional annotation further inform the biology of contigs. BinaRena enables convenient toggling among these characteristics. The user may further specify data transformation, data range, and color map (for both discrete and continuous data) in the interface. The program implements multiple transformation methods to deal with various types and distributions of biological data, including square and cube (root), logarithmic and exponential, logit and arcsine, and ranking, all of which can be easily triggered from a dropdown menu. The user may move and zoom the plot with mouse and/or keyboard, just like navigating a typical digital map. All panels can be uncollapsed to screen corners to minimize distraction during data exploration.

The contig data can be provided as one integrated data table, or as multiple tables or mappings sequentially appended to the same dataset, which improves flexibility and lowers the challenge in preparing input files. BinaRena accepts four data types: numeric, categorical, feature set, and descriptive. The feature set data type, provided as comma-separated strings, lets the user specify gene content of each contig, annotated either for general purpose (such as KEGG [37] Ontology, or KO) or to address specific research questions (such as phylogenetic markers [38], antimicrobial resistance genes, mobile genetic elements, or members of a specific metabolic pathway).

BinaRena further lets the user specify feature groups, defined by a list of member features that constitute a group. Then the program can calculate the completeness and redundancy (a.k.a., contamination) of user-selected contig groups in real time. This significantly improves the convenience and flexibility to assess the quality of a putative bin with or without a specific biological question, as in contrast to currently adopted protocols which are usually performed when bins are already defined. It should be noted, however, that BinaRena does not consider marker gene collocation as CheckM does [38]; therefore, their results are not identical, albeit highly correlated (Fig. S1); thus, the former can serve as a first-pass check, while the latter is still recommended post-binning.

BinaRena offers a variety of controls for exploring the metagenomic dataset. Contigs can be selected by mouse clicking or by drawing a polygon to contain multiple contigs. The selection is retained as the aesthetics are toggled, allowing the user to explore the same contigs of interest using different data. With a single keystroke or button, the selected contigs can be highlighted using choice of colors to indicate user interest, they can be “masked” such that they are both hidden from the plot and excluded from subsequent manipulations and calculations, and they can be “focused” such that only them but no other contigs are visible, which facilitates user concentration. These operations can be “undone” to revert to previous status. Contigs can be searched based on their numeric and categorical properties as well as features they carry.

The properties of selected contigs are summarized in a side panel by user-specified methods that make most sense for the nature of data. Examples are as follows: “length” is the sum of contig lengths. “GC” is the average of GC contents weighted by length. The category (such as taxonomic group) of multiple contigs is determined by the majority rule, optionally weighted by length, with the fraction annotated as a suffix (e.g., “Firmicutes (80%)”). Aside from the scatter plot, there is a mini-interactive histogram displaying the distribution of a user-designated numeric property (such as coverage) of the selected contigs. The user can use mouse dragging to filter the contigs by data range (such as a peak of coverage values). This function is useful for refining a contaminated bin.

BinaRena provides handy controls for assigning contigs to bins that represent putative MAGs. The user can create a binning plan de novo or edit binning plans computed by external programs. Bins are displayed in an interactive table and summarized by their total length and abundance per sample. Using one keystroke or button, the user can add or remove selected contigs to or from individual bins. BinaRena implements two algorithms for the evaluation and comparison of binning plans. It calculates the silhouette coefficient [39] to assess the confidence of assigning contigs to individual bins. The results can be visualized instantly as color depth to provide an intuitive view of the bin confidence profile. The program also calculates the adjusted Rand index [39] to assess the consistency between pairs of binning plans. Both metrics are widely used in cluster analysis. However, BinaRena’s ability to calculate them during exploration significantly supports the user effort.

BinaRena can output various types of files to support the sharing and reporting of analysis results. The binning plans and the contig data of individual bins can be exported as TSV table files. The scatter plot along with legends and axes can be exported as a PNG bitmap image or an SVG vector image for post-processing and publication. Critical information of a run, including filtering thresholds and calculation results, is logged and can be exported as a text file. At any moment during a run, a “checkpoint” can be exported as a JSON file, which can later be loaded back to resume the same interactive view of the dataset. These features facilitate the reproduction of BinaRena results.

Besides the main program, BinaRena provides multiple Python scripts to aid data preparation. They include utilities to count *k*-mers from contig sequences, followed by dimensionality reduction using PCA, t-SNE, and UMAP to infer coordinates of contigs in a scatter plot. These three analyses were enabled by calling the common Python libraries scikit-learn and umap-learn. They also include utilities to convert common metagenomics tool outputs into the table format. Examples are SPAdes [40] and MetaHIT [41] assemblies, GTDB-tk [42] lineage strings, Kraken [43] taxonomic assignments, GFF-formatted genome annotations, and CheckM [38] marker gene maps. The software’s documentation includes a tutorial demonstrating an entire workflow from raw sequencing data to processed input files for BinaRena. A video introduction to BinaRena’s functionality is provided in Data S1.

### Comparison with existing tools

Here, we review multiple existing tools for interactive visualization of metagenomic contigs and compare them with BinaRena. Anvi’o [28] is an integrated multi-omics platform that is most known for an interactive sector graph depicting sequence composition and per-sample abundance of contigs with the ability to add customizable layers, allowing users to explore classification, evolutionary, and functional capacity patterns of the dataset. This visualization method is highly effective for exploring contig distribution among samples but less so for the relationships among contigs. The complexity in setting up a server and executing command-line workflows to prepare for visualization may challenge nontechnical users. The visualization tool ICoVeR [29] is for user-guided refinement of existing binning plans. It renders a line graph depicting per-sample abundance of a co-assembly, as well as other numeric metrics. It supports generation of scatter plots and histograms using several clustering and ordination algorithms; however, these plots are for exploring variables instead of contigs. The ggKBase [30] workflow is suitable for manual binning. It employs an interactive wheel for selecting taxonomic groups and a histogram for selecting metric ranges (also supported by BinaRena). Collectively, BinaRena’s interactive scatter plot of contigs does not overlap with Anvi’o, ICoVeR, and ggKBase but instead may serve as a complement to current metagenomics workflows that use these tools. BusyBee Web [31] is a web server that performs the entire binning workflow. Its interface displays contigs as a scatter plot, which is mainly for exploring pre-computed (by the server) bins, and does not support complex contig and bin operations. Likewise, it displays CheckM-calculated bin quality metrics, rather than evaluates bin quality interactively as BinaRena does. We would like to note that BusyBee Web’s predecessor, VizBin [36], was the original source of inspiration to the development of BinaRena. To our knowledge, Elviz [32] is the most comparable existing tool to BinaRena. The Elviz server is integrated into the JGI portal, which provides convenience but also imposes restrictions to the user. It emphasizes on assessing the taxonomy and functions of contigs, but it can be repurposed for editing bins. An itemized comparison of BinaRena and Elviz is provided in Table S1, showing that the former is notably more feature rich. Finally, an obvious advantage of BinaRena compared with all these tools is the ease of deployment. In summary, we believe that BinaRena is a unique bioinformatics tool for the task it aims to achieve.

## Results

### Exploring microbial populations responsible for nutrient cycling in the Maquia peatland

Extensive tropical peatland formations have been reported in the Pastaza-Marañon basin in the Peruvian Amazon [44], among which the “open peatland” constitutes a unique category which is devoid of trees but dominated by arbustive vegetation [45]. Given their role sequestering organic carbon in their soils and to understand their microbial functions [46], we sampled an open peatland (Maquia: MAQ) for metagenomic evaluation. Quality-filtered reads from all six samples were co-assembled, and subsequent contigs were binned using automatic binners MaxBin [16] and MetaBAT [17], yielding 251 and 345 total bins, respectively. The two results were consolidated using DASTool [18], yielding 276 total bins. These bins span 25 phyla. BinaRena was used to render placement of contigs from this assembly (contigs ≥ 2000 bp) with t-SNE based on tetranucleotide frequency (Fig. 1). The contig aesthetics (size, color, and opacity) are associated with common properties such as contig length, taxonomic classification, and abundance in a sample from depth = 10 cm (see “Materials and methods”). This initial view revealed that many contigs are associated with taxonomic groups from Proteobacteria, Acidobacteria, and Actinobacteria. Additionally, Methylomirabilota, Desulfobacterota, and Nitrospirota form two distinct tight clusters. BinaRena’s polygon tool was used to select a distinct cluster of contigs, representing multiple populations of Nitrososphaeria. Classified under phylum Thermoproteota, Nitrososphaeria are ubiquitous terrestrial ammonia-oxidizing archaea [47].

**Fig. 2:**
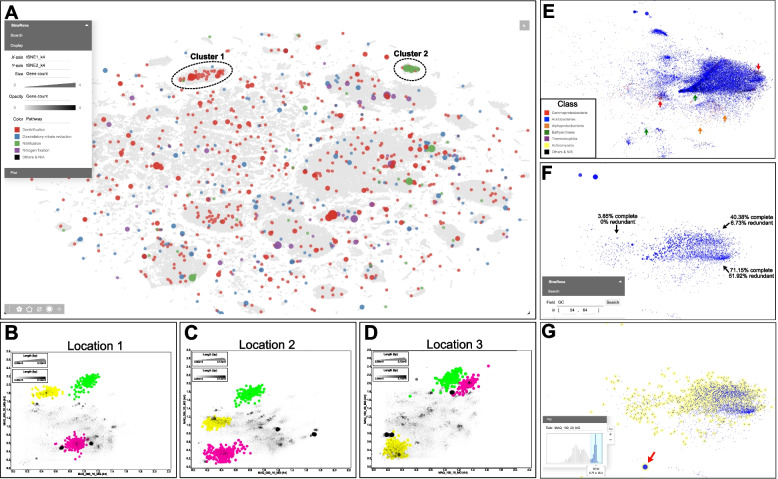
Distribution of nitrogen cycling genes and exploration of *Sulfotelmatobacter* populations in the MAQ dataset. **A** An overview of the entire assembly. The *x*- and *y*-axis represent t-SNE embeddings based on tetranucleotide frequencies. Marker size (square root) and opacity are proportional to the number of KOs assigned to each contig that is associated with the previously described nitrogen pathways, and the color represents that pathway. **B**–**D** BinaRena-exported SVG images (rasterized) depicting the change in abundance of the Streptosporangiales MAG (pink), Thermoanaerobaculales MAG (yellow), and Nitrososphaerales (green). The *x*-axis represents coverage at depth 10 cm, while the *y*-axis represents coverage at depth 20 cm. The only edits to raw files generated by BinaRena were an increase in font size, changes to legend text, and resizing of the plot area to decrease white space. **E** Subset of contigs classified as Koribacteraceae or were assigned to one of the five *Sulfotelmatobacter* MAGs is plotted using t-SNE (*k* = 6) and colored by class. The size is proportional to the contig length, and opacity is the cube for coverage in location 3, depth 20 cm. Arrows are pointing at regions with contigs of potential contamination. **F** Contigs in E that have been filtered to a range of 54–64% GC (inset). All other aesthetics remain the same. **G** Contigs highlighted in yellow were selected based on high abundance in location 3, depth 20 cm using the histogram (inset). All other aesthetics remain the same except for size which is proportional to the cube root of the amount of sulfur genes found on contigs. The red arrow is pointing at the potentially missing contig from the *Sulfotelmatobacter* MAG

Genome-resolved metagenomics is widely used to understand potential biological mechanisms within an ecosystem and its distribution across the community. Nitrogen cycling within tropical peatlands is relatively understudied, yet there is evidence that it is closely interconnected with the release of greenhouse gasses from these environments [48, 49]. Here, we used BinaRena to explore the distribution of nitrogen cycling genes involved in pathways such as dissimilatory nitrate reduction, denitrification, nitrification, and nitrogen fixation. Visualization in BinaRena supports quick identification of contigs containing genes for the previously listed pathways of interest (Fig. 2A). The distinct cluster of Nitrosophaeria contigs contains copies of both the *nosZ* and *nirK* (cluster 1). Additionally, a different cluster of Nitrosophaeria contigs (cluster 2) was found with copies of *amoABC*, which is consistent with culture-based studies [50] of Nitrososphaeria as an ammonia oxidizer. Overall, BinaRena assisted in identification and quantification of the importance of Nitrosophaeria potentially in the MAQ nitrogen cycle.

To understand how there might be differences in nitrogen cycling populations across the 100-m transect, we focused on two high- and one medium-quality bins (defined following [1]) inferred by DASTool that were identified as capable of dissimilatory nitrate reduction (Streptosporangiales: 96.77% complete/3.83% contaminated, calculated by CheckM, same below) and denitrification (Nitrososphaerales: 76.79%/0.93% and Thermoanaerobaculales: 94.12%/4.2%) (Fig. 2B–D). Contigs in the Thermoanaerobaculales MAG form a distinct cluster in the BinaRena graph representing the coverage profile at both 10 cm and 20 cm depths at location 1 (Fig. 2B). This MAG is predicted to carry out nitrite reduction (*nirK)*, a suboxic process [51], and likely why we find it at a higher abundance at 20 cm in the soil. However, abundance of this MAG progressively decreases in location 2 and then location 3 (17.92 × to 4.41 × to 0.07 ×). Conversely, the Streptosporangiales MAG is found at very low abundance at both locations 1 and 2 but becomes abundant at 10 cm (5.2 ×) and 20 cm (14.12 ×) depths in location 3. While we observed spatial variation in the abundance of both the Thermoanerobaculales and Streptosporangiales MAGs, there was minimal variation detected in Nitrososphaerales. The Nitrososphaerales was the most abundant MAG across all three locations (31.83 × , 33.94 × , 18.79 ×) at 20 cm depth. It is interesting to consider what environmental factors are contributing to the change in abundance of both the Thermoanaerobaculales and Streptosporangiales MAGs. However, this falls outside the scope of this study but demonstrates BinaRena’s utility in hypothesis generation.

BinaRena is capable of restructuring contig placement, expediting identification of dynamics between populations while also supporting MAG refinement by identifying potentially misplaced contigs (and genetic potential). The recently discovered genus of *Sulfotelmatobacter* is potentially capable of carrying out dissimilatory sulfite or sulfate respiration [52], with implications for organic matter decomposition and greenhouse gas production. The *Sulfotelmatobacter* MAGs recovered using the three automated binners exhibited relatively high contamination and/or low completeness and lacked genes involved in sulfur metabolism (Fig. S2). To improve MAG quality, we selected all contigs associated with these five MAGs and contigs that were classified as Koribacteraceae and then subsequently visualized using the t-SNE at *k* = 6 (Fig. 2E). We found most contamination (from contig-to-MAG selection) coming from contigs classified as Alphaproteobacteria (280), Gammaproteobacteria (61), and Bathyarchaeia (15), which were selected and removed. After removal, we reassessed the distribution of contigs and selected those that fell within a tight range of GC content (54–62%, based on MAGs generated from the automatic binners, as well as what has been previously published on this group [52]) and were removed using BinaRena.

There were three visually distinct clusters of contigs, but binning these resulted in either low completeness or high contamination (worse than the automated binners) (Fig. 2F). To better account for differences between populations, we further focused on contig abundance across location and depth. Using BinaRena’s interactive histogram, we separated contigs that were at high abundance in location 3 at 20 cm depth (*Sulfotelmatobacter* are predicted anaerobes, and the MAGs from the automated binners were the most abundant in location 3 at 20 cm) (Fig. 2G). This retained 1034 contigs with a total size of 3.98 Mbp and an average of 13.95 × coverage. Furthermore, the completeness and contamination of this MAG marginally increased to 58.55% and 3.41%, respectively. This MAG comprised 78.66% of the MetaBAT no. 229 bin and 99.4% of the Maxbin no. 235_sub bin from DASTool. While quality only slightly increased, the MAG now contained genes for dissimilatory sulfate respiration (*dsrAB*) (Fig. 2G, red arrow). These genes were previously found by MaxBin (no. 235) but were removed by DASTool. To support their placement within this bin, both *dsrA* and *dsrB* genes were blasted, and the top ten matches belong to an uncultured sulfate-reducing organism. This 2139-bp-long contig does not cluster with the rest of the contigs (based on t-SNE *k* = 6) but has consistent coverage across metagenomes with the exception of MAQ_050_10_MG (Fig. S2F).We suggest that contig misplacement by the automated binners, due to challenges with binning contigs ≤ 2000 bp [21], could be caused by the elevated abundance of this contig within MAQ_050_10_MG. This elevated coverage might indicate natural variation within *Sulfotelmatobacter* populations, such as copy number, which is undetectable by automated binners. By implementing both targeted classification, GC% and depth metrics (for what is known about *Sulfotelmatobacter*), we were able to recover a more complete representation of the ecosystem. In summary, BinaRena directly facilitated the curation of this MAG, which prior to human intervention lacked biological significance.

### Separating closely related pathogenic microbes in traveler’s diarrhea gut metagenomes

Travelers’ diarrhea (TD) is an intestinal disorder caused by infection during traveling [53]. Identification of infectious agents is of epidemiological importance but challenging due to the diverse and unpredictable pathogenic profiles [54]. In a previous study, Zhu et al. studied the metagenomes of a TD cohort, and discovered multiple putative pathogens, some of which were confounded by closely related organisms in the same sample [33]. The current study provides a revisit to the question using the BinaRena program, as exemplified by two difficult samples.

**Fig. 3:**
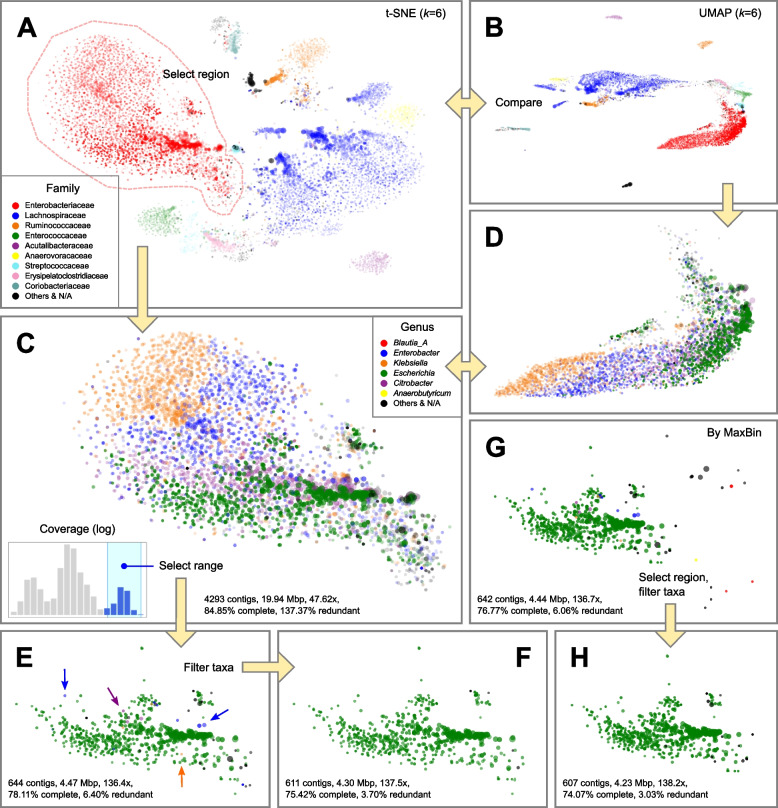
Extraction of a pathogenic *Escherichia coli* MAG from several closely related organisms in the metagenome from the gut of a travelers’ diarrhea patient (sample no. 76, with 2.92-Gbp raw reads, 10,910 contigs totaling 69.5 Mbp). Marker size (radius) is proportional to the cube root of contig length. Marker opacity is proportional to the cube root of contig coverage. Colors were assigned to the most abundant taxa in the sample. The assembly data (**A**, **B**) was explored using alternative dimensionality reduction methods (t-SNE for **A**, **C**, **E**–**H**; UMAP for **B**, **D**, both based on *k*-mer (*k* = 6) frequencies). A distinct blob of Enterobacteriaceae contigs (**A**, dashed line) were selected (**C**, **D**) and filtered based on its coverage profile (**C**, inset), resulting in a putative *E. coli* bin (**E**), which was further filtered by taxonomy (exemplified by arrows in **E**) to improve purity (**F**). In parallel, the corresponding bin inferred by MaxBin (G) was filtered by spatial pattern and taxonomy to retain a purer bin (**H**)

Sample no. 76 was characterized by the co-infection of multiple putative pathogens under the genera of *Escherichia*, *Enterobacter*, *Klebsiella*, and *Citrobacter*, all belonging to the family of Enterobacteriaceae [33]. Automated binning algorithms, dependent on sequence similarity, struggle with the task of assigning contigs to appropriate genomes, particularly when there is high evolutionary proximity between populations [38]. The relatively shallow sequencing depth (2.92-Gbp raw reads in total) further adds to the difficulty in recovering MAGs of reasonable quality. We performed visual observation of the assembly in BinaRena, showing that t-SNE and UMAP at *k* = 6 provided the most apparent visual consistency between contig clustering pattern and taxonomic assignment (Figs. 3A, B, S3). By cross-comparing the two views, we selected a cluster of contigs that were mainly assigned to Enterobacteriaceae using BinaRena’s polygon selection tool. BinaRena reported that this cluster contains 4293 contigs totaling 19.9 Mbp, with an average coverage of 47.62 × (weighted by contig length). A total of 98.42% of the length was assigned to family Enterobacteriaceae. By assessing CheckM’s Enterobacteriaceae-specific marker genes (*n* = 297), BinaRena determined that this cluster has completeness = 84.85% and contamination = 137.37%, indicating the presence of multiple genomes (Table S2, same below). Coloring by genus clearly showed that this cluster contains contigs assigned to all four pathogenic genera, which are visually distinguishable but hard to separate (Fig. 3C, D). The histogram of contig coverage showed several peaks, again implicating the presence of multiple genomes (Fig. 3E, inset). We separated the high-end peak by mouse-dragging six bins out of 20 in the interactive histogram (Fig. 3E, inset). This retained 644 contigs (4.47 Mbp, 136.4 ×), with 95.87% of its length assigned to genus *Escherichia* (Fig. 3E). They are 78.11% complete and 6.40% contaminated. Next, we used BinaRena’s search tool to identify and remove non-Gammaproteobacteria contigs, and contigs assigned to the other three pathogenic genera (*Enterobacter*, *Klebsiella*, and *Citrobacter*), which are presumably contaminations. This left 611 contigs (4.30 Mbp, 137.5 ×), with completeness = 75.42% and contamination = 3.70%, which we consider as a putative MAG of *Escherichia* (Fig. 3F), a taxon containing common causative pathogens for TD [54].

We then explored binning plans generated by automatic binners (MaxBin, MetaBAT, and DASTool). BinaRena’s information panel indicates that MaxBin’s bin no. 001 (642 contigs, 4.44 Mbp, 136.7 × , 76.77% complete, 6.06% contaminated) has the highest consistency with the manually isolated *Escherichia* MAG as detailed above (97.08% of the latter length was shared between the two; Jaccard index = 0.901) (Fig. 3G). However, this bin contains multiple “outlier” contigs that are approximate to other clusters indicated by both *k*-mer signature and taxonomic assignment, implicating contaminations (Fig. S4A). Therefore, we manually refined this bin by removing the “outlier” contigs using polygon and then by taxonomic filtering as detailed above (Fig. 3H). The curated bin has 607 contigs (4.23 Mbp, 138.2 ×), with completeness = 74.07% and contamination = 3.03%, and has higher consistency with the manually extracted MAG (Jaccard index = 0.952). In parallel, MetaBAT recovered a bin (403 contigs, 88.89% complete, 30.98% contaminated) that was a mixture of a portion of *Escherichia* contigs and a clearly separate cluster of contigs that were assigned to genus *Faecalibacterium*, a common commensal component of the gut microbiota [55]. This observation points to putative chimerism (Fig. S4B). Finally, the ensemble method DASTool kept the MetaBAT bin, and stripped the shared part from the MaxBin bin, leaving only 304 contigs (39.06% complete, 2.36% contaminated) (Fig. S4C), a result that is suboptimal.

In parallel, we investigated sample no. 50076, characterized by the co-infection of multiple *Escherichia coli* strains [33]. An overview of the assembly in BinaRena supports a clear *E. coli* dominance pattern (Fig. S5A). Among the 27 bins inferred by MaxBin, seven have more than 50% of their total length assigned to genus *Escherichia*; however, four of them are less than 2% complete as evaluated by BinaRena using CheckM’s *E. coli*-specific marker genes (*n* = 1628). The remaining three have a total length between 1.2 and 1.7 Mbp, average coverage between 2100 and 3100 × , completeness between 18 and 41%, and contamination below 0.5% (Table S3). These metrics indicate that they are highly incomplete *E. coli* genome; the relatively even coverage values and the a priori knowledge that *E. coli* genomes are usually 4.5–5.5 Mbp long [56] led us to postulate that these bins may be parts of one *E. coli* genome. Similarly, MetaBAT inferred two *E. coli* bins (one was retained by DASTool), each of which also seemingly partial (Table S3). These results expose the limitation of automatic methods which often fail to resolve strain-level variation [19], from resolution issues with sequence/coverage metrics, and produce either fragmented/incomplete or “mega” bins. Therefore, we resorted to de novo binning using BinaRena. Similar to the method described above, we first selected the cluster of contigs that were dominantly assigned to *Escherichia*, with 554 contigs, 6.89 Mbp, 1939 × , 98.40% complete, and 4.91% contaminated (Fig. S5B). These metrics indicate that there may be secondary *E. coli* genomes mixed in it, which is also evident from the multimodal pattern of the contig coverage histogram (Fig. S5A, inset). Likewise, we selected the top five bins out of 20 (coverage ≥ 1110 ×), resulting in 309 contigs, 5.07 Mbp, 2515 × , 98.03% complete, and 1.29% contaminated (Fig. S5C). Compared with the automatically inferred bins, this bin notably better represents a complete *E. coli* genome that dominated the patient’s gut among other less abundant *E. coli* strains.

### Systematic improvement of binning results using the CAMI2 marine metagenomes

We further demonstrated that BinaRena can help to efficiently and systematically improve bin quality of an entire dataset. For this purpose, we used the synthetic marine metagenomic dataset from the 2nd CAMI challenge, a gold standard for assessing the performance of metagenome binning algorithms [19]. A researcher, with no prior experience with the CAMI2 dataset, worked on each binning plan generated by MaxBin, MetaBAT, and DASTool. Briefly, contigs associated with each bin were highlighted, and then the *x*- and *y*-axes were toggled (between both PCA and coverage profiles) to identify potential misplaced contigs. The binning plans pre- and post-curation were evaluated using the silhouette coefficient and the adjusted Rand index (ARI), both calculated in the BinaRena interface, and the completeness and contamination scores calculated by CheckM (outside BinaRena). It should be noted that the researcher was agnostic about these metrics during curation, as this functionality was not implemented until after the curation process.

**Fig. 4:**
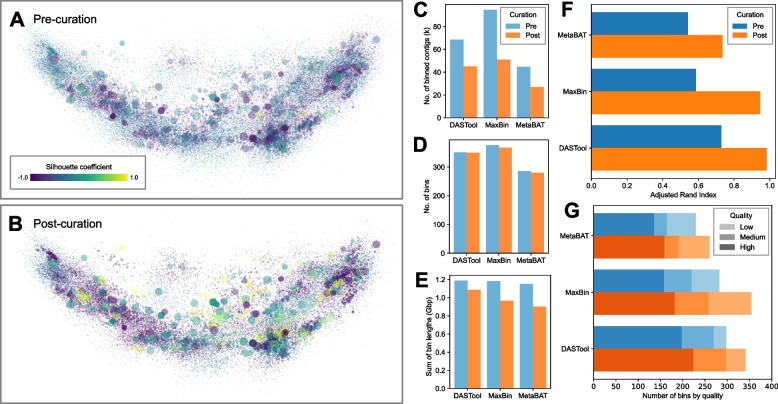
Curation of entire binning plans on the CAMI2 marine dataset. **A** and **B** The gold standard assembly was visualized using PCA on tetranucleotide frequencies, showing DASTool-binned contigs pre- (**A**) and post- (**B**) curation using BinaRena. Contigs are colored by the silhouette coefficient calculated by BinaRena. Marker size (radius) is proportional to the cube root of contig length. Marker opacity is proportional to the 4th power root of average contig coverage of 10 samples. **C**–**G** Metrics of three binning plans (generated by DASTool, MaxBin, and MetaBAT, respectively) pre- and post-curation. **C** Total number of contigs in bins. **D** Total number of bins. **E** Total length of contigs in bins. **F** Adjusted Rand index between each binning plan and the ground truth as calculated by BinaRena. **G** Numbers of high-, medium-, and low-quality MAGs, defined following [1] based on CheckM-inferred completeness and contamination scores. Specifically, high quality: ≥ 90% complete, < 5% contaminated; medium quality: ≥ 50% complete, < 10% contaminated; and low quality: < 50% complete, < 10% contaminated. Bins that do not match any catalog (i.e., ≥ 10% contamination) are excluded

Comparative analysis showed that after curation using BinaRena, the binning plan was visibly more consistent with the clustering pattern of contigs, as indicated by silhouette (Fig. 4A, B). BinaRena’s capability of calculating and visualizing silhouette as the user modifies the binning plan is useful for curation (although not used in this analysis). A portion of contigs were filtered out from the bins during curation (Fig. 4C), accompanied by infrequent deletion of entire bins (Fig. 4D). The removed contigs were usually small; therefore, the loss in the total bin length was moderate (Fig. 4E). As evaluated by ARI, the post-curation binning plans are notably more consistent with the ground truth genome assignment, as compared to the pre-curation ones (Fig. 4F). For example, ARI of the DASTool-inferred bins increased from 0.729 to 0.982, suggesting that the latter are a nearly perfect subset of the true genomes. The quality of curated bins following the adopted standard [1] suggested a notable increase in the number of all publishable MAG categories (high, medium, and low quality) (Fig. 4G). These results indicate a substantial improvement in the overall quality of binning plans using BinaRena.

## Discussion

We developed BinaRena to support researchers to more effectively and comprehensively visualize and operate on metagenomic datasets. In this work, we have demonstrated that BinaRena can assist human researchers to quickly identify patterns at the community scale with taxonomic and functional relevance in addressing biological questions while also isolating relevant MAGs from the background. In addition, we have illustrated issues that can arise from solely using automated binners, and that the use of BinaRena can aid in both identification and improvement from abovementioned issues. Even when used as a de novo binner, BinaRena could yield MAGs with comparable or even better quality than the best result of several automatic binners. Meanwhile, it is effective in curating binning plans computed by automatic binners and achieving improved quality of the recovered MAGs.

BinaRena’s ease of operation and versatility facilitate metagenomic analysis for both novice and expert users. Being a dependency-free, client-end single web page, BinaRena is among the easiest of all bioinformatics tools in terms of deployment and use. This characteristic also grants potential for effortless integration of BinaRena into current metagenomics workflows. In contrast to the simplicity of start-up, BinaRena has rich features that permit complex operations on metagenomic data. Meanwhile, the program’s deliberate user interface (UI) design provides an efficient and comfortable workspace for human operators, and this is of importance because the exploration of complex data requires labor and concentration. Noting its high customizability, we envision that BinaRena may also be useful in other research tasks involving classification, clustering, and/or ordination, although further work is needed to establish this point.

While being a useful tool for microbiome researchers, BinaRena is not meant to replace automatic binners. The analysis is highly impacted by human behavior, which could introduce bias. Careful documentation and reasoning (as done in this work) ensure reproducibility of one analysis, but do not warrant generalization of the protocol to other cases. We recommend the adoption of BinaRena in addition to automatic workflows, the results of which are also useful input for BinaRena, as demonstrated above. On top of all, BinaRena is suitable for data overview, hypothesis generation, and sanity check of analysis results. Beyond, BinaRena lets the researcher focus on individual MAGs that are of high relevance to the research topic. Lastly, BinaRena can help if the research goal is to maximize the quality of an entire binning plan, although this would require significant human labor.

The pursuit of decoding complex metagenomic data and deconvoluting them into original organismal entities is of central importance yet so far challenging. BinaRena represents progress in one direction of multiple to the solution of this problem. Future efforts should be attributed to better integration of algorithms and human factors into a semi-supervised workflow that simultaneously achieves high accuracy, interpretability, and reproducibility.

## Conclusions

We present BinaRena, a software tool for interactive visualization and operation of metagenomic contigs, to facilitate discovery of biological patterns and recovery of metagenome-assembled genomes (MAGs). Engineered with a strong focus on human factors, it lets the user observe various characteristics of large metagenomic datasets, and efficiently manipulate contig-bin assignments, as well as assess contig group properties and MAG quality metrics which can help with human decision-making. BinaRena effectively complements algorithmic workflows and benefits researchers of all technical levels in various types of microbiome studies.

## Materials and methods

### The Maquia peatland dataset

The Maquia peatland (MAQ) metagenomes were sampled in the Yanayacu-Maquia Conservation Concession, Peru (6°22′ S 74°53′ W), in October 2015. Six samples were collected from soil cores at three spatial intervals 50 m apart at depths of both 10 and 20 cm. DNA extraction was performed using the MicroSoil kit (QIAGEN, CA, USA) following the general protocol proposed by the earth microbiome project [57]. High-throughput sequencing was performed on an Illumina NovaSeq platform at JGI, NM, as part of their 2015 Community Sequencing Program. Sequencing data were processed using Trimmomatic v0.40 [58]. Quality-trimmed sequencing data were deposited at JGI for MAQ (Ga0314862-Ga0314867).

### The travelers’ diarrhea dataset

The travelers’ diarrhea (TD) dataset [33] contains 29 metagenomic samples, sequenced from fecal materials of individuals who traveled from the USA to Mexico or India between 2005 and 2010. Twenty-two subjects developed TD but were tested negative for common TD pathogens, implicating the presence of novel pathogens, whereas the remaining seven were healthy. We reanalyzed the published sequencing data (NCBI PRJNA382010) using currently adopted workflows (see below). The metagenomes were assembled separately due to the lack of shared pathogenic profiles. Two samples, no. 76 and no. 50076, which were shown to contain closely related pathogens [33], were selected for demonstrating BinaRena’s functionality in this study.

### The CAMI2 marine dataset

The 2nd CAMI challenge [59] marine metagenomes (Illumina) gold standard assembly (GSA) was retrieved from PUBLISSO (https://doi.org/10.4126/FRL01-006425521). It contains 10 samples, simulated to represent microbial communities at different seafloor locations of a marine environment. Contigs that are at least 2000 bp, totaling 159,957 contigs, 1.816 Gbp, were used for binning. The per-sample abundance values were used in this study to assist manual curation of binning plans. The ground truth genome assignments were retrieved from the CAMI GitHub repository (https://github.com/CAMI-challenge), under the following: second_challenge_evaluation/tree/master/binning/genome_binning/marine_dataset/data/ground_truth/.

### Assembly and automatic binning of metagenomic datasets

Both MAQ and TD metagenomes were co-assembled using MegaHit v1.2.9 [41] using the “–meta” preset. Resulting contigs were filtered based on a minimum length of 2000 bp and an average coverage greater than 1 × over 90% of the contig length. Metagenomic reads from each sample were mapped back to contigs using Bowtie2 v2.3.5.1 [60], and depth profiles were generated using the “jgi” script provided in MaxBin2 v2.2.7 [16]. For all four datasets (MAQ, TD, and marine), filtered contigs were binned using MetaBAT2 v2.2.15 [17] and MaxBin2 [16] with default settings. Results from these binning plans were consolidated using DASTool v1.1.3 [18]. Resulting MAGs were assessed for quality using CheckM v1.1.3 [38], and GTDB-tk v1.7.0 [42] was used to determine taxonomy of all bins. Bins with both completeness and contamination scores equal to zero according to CheckM were not investigated.

### Data preparation for BinaRena

Length, coverage, GC content, and *k*-mer frequencies (*k* = 4, 5, and 6) of individual contigs were calculated using previously published scripts [16, 61]. The *k*-mer frequency profiles were subject to three mainstream dimensionality reduction methods: PCA [62], t-SNE [63] (implemented in scikit-learn v1.0.2), and UMAP [64] (implemented in umap-learn v0.5.3). Prior to the t-SNE analysis, the dataset was processed using PCA to retain 50 dimensions. The Barnes-Hut approximation [65] was used to accelerate the t-SNE calculation, following previous works [36, 66, 67]. The UMAP analysis was also based on the same 50 PCA-reduced dimensions. Contigs from the MAQ and TD datasets were annotated using KofamScan v1.3.0 [68] against KOfam release 2022–03-01. Taxonomy was assigned to contigs using Kraken2 v2.1.2 [43] with default settings against the GTDB release 202 [69].

### Availability and requirements

- Project name: BinaRena
- Project home page: https://github.com/qiyunlab/binarena
- Operating system(s): Platform independent
- Programming language: JavaScript
- Other requirements: A modern web browser (Chrome, Firefox, Safari, Edge, etc.)
- License: BSD-3-clause

### Supplementary Information

**Additional file 1:** **Fig. S1.** Correlation between completeness (A) and redundancy (contamination) (B) values calculated by BinaRena and CheckM. A total of 596 bins recovered by MaxBin and MetaBAT from the MAQ dataset were evaluated. The CheckM marker gene set for domain Bacteria was used, which contains 104 genes arranged in 58 sets. The regression line (black) is plotted in each panel. The Pearson’s correlation coefficient (*r*) and its *p*-value are marked under the plot. **Fig. S2.** *Sulfotelmatobacter* MAGs identified by three automatic binners. Scatter plots were defined by t-SNE on *k*-mer (*k* = 6) frequencies. Marker size (radius) is proportional to contig length. Marker opacity is proportional to the cube root of contig coverage in location 3 at depth of 20cm. A. MaxBin’s result. B, C. MetaBAT’s results. D, E. DASTool’s results which are both subsets of MaxBin results. D came from a bin with high redundancy (contamination) (40.89%) and only classified to the family level (Koribacteraceae) and E is a subset of panel A. F. Multi-panel scatter plot representing contig coverage across metagenomes for curated Sulfotelmatobacter MAG. Gray circles represent contigs without *dsrAB*, while the red circle represents the potentially misplaced contigs with *dsrAB*. **Fig. S3.** Various views of the TD metagenome #76. Three dimensionality reduction methods, PCA, t-SNE, and UMAP, were applied to *k*-mer frequency profiles with *k* = 4, 5, and 6. In addition, the coverage (log) was plotted against GC content and contig length (log). Marker size (radius) is proportional to the cube root of contig length. Except for the last two plots (in which contig coverage is the *y*-axis), marker opacity is proportional to the square root of contig coverage. Colors are assigned to the top nine most abundant families. The color codes are identical to that of Fig. 3A, B. **Fig. S4.** Comparison of an *Escherichia* MAG identified by three automatic binners. Scatter plots were defined by t-SNE on *k*-mer (*k* = 6) frequencies. Marker size (radius) is proportional to the cube root of contig length. Marker opacity is proportional to the square root of contig coverage. A. MaxBin’s result (see also Fig. 3G), which has the highest consistency with the manually identified MAG (Fig. 3F). B. MetaBAT’s result (also DASTool’s primary result), which contains a proportion of the *Escherichia* contigs plus a separate contig cluster assigned to genus *Faecalibacterium* (dashed circle). C. DASTool’s secondary result (equivalent to the MaxBin bin excluding the MetaBAT bin). **Fig. S5.** Recovery of a pathogenic *Escherichia coli* MAG from TD sample #50076 (19.82 Gbp raw reads, 9,816 contigs totaling 62.2 Mbp), which contains multiple *E. coli* strains. Scatter plot was defined by t-SNE on *k*-mer (*k* = 6) frequencies. Marker size (radius) is proportional to the cube root of contig length. Marker opacity is proportional to the cube root of contig coverage. Colors were assigned to the top 14 most abundant genera in the sample. A. View of the entire assembly. A cluster of contigs mainly assigned to *Escherichia* was selected (dashed polygon). B. The selected cluster of contigs. Its coverage profile exhibits a multi-modal pattern (inset of A). Therefore, the top five out of 20 bins of the histogram were retained. C. The retained contigs, which represent a putative *E. coli* MAG. **Table S1.** Comparison of functionality of BinaRena and Elviz. **Table S2.** Metrics of contig clusters / bins in TD sample #76 calculated by BinaRena. **Table S3.** Metrics of contig clusters / bins in TD sample #50076 calculated by BinaRena.

**Additional file 2.** Data S1.

## Data Availability

The source code of BinaRena is publicly available at: https://github.com/qiyunlab/binarena, under the BSD-3-clause license. The data and scripts presented in this manuscript are publicly available at https://github.com/pavia27/BinaRena-manuscript.

## Abbreviations

ARI: Adjusted Rand index
CAMI: Critical Assessment of Metagenome Interpretation
MAG: Metagenome-assembled genomes
MAQ: Maquia peatland
PCA: Principal component analysis
TD: Travelers’ diarrhea
t-SNE: T-Distributed stochastic neighbor embedding
UMAP: Uniform manifold approximation and projection
